# Hydrogeochemical Characteristics and Conceptual Model of the Geothermal Waters in the Xianshuihe Fault Zone, Southwestern China

**DOI:** 10.3390/ijerph17020500

**Published:** 2020-01-13

**Authors:** Xiao Li, Xun Huang, Xin Liao, Yunhui Zhang

**Affiliations:** 1 State Key Laboratory of Geological Disaster Prevention and Environmental Protection, Chengdu University of Technology, Chengdu 610059, China; lixiao@cdut.edu.cn (X.L.); huangxun248@163.com (X.H.); 2 Faculty of Geosciences and Environmental Engineering, Southwest Jiaotong University, Chengdu 611756, China; xinliao@swjtu.edu.cn

**Keywords:** geothermal water, water-rock interaction, geothermometry, recharge origin, Xianshuihe fault zone

## Abstract

Abundant geothermal waters have been reported in the Yalabamei, Zhonggu, Erdaoqiao, and Yulingong geothermal areas of the Xianshuihe Fault Zone of western Sichuan, southwestern China. This study focused on the hydrogeochemical evolution, reservoir temperature, and recharge origin of geothermal waters using hydrochemical and deuterium-oxygen (D-O) isotopic studies. Shallow geothermal waters represented by geothermal springs and shallow drilled water wells are divided into two hydrochemical groups: (1) the Ca–Na–HCO_3_ type in the Erdaoqiao area, and (2) Na–HCO_3_ in other areas. Deep geothermal waters represented by deep drilled wells are characterized by the Na–Cl–HCO_3_ type. The major ionic compositions of geothermal water are primarily determined by the water–rock interaction with silicate and carbonate minerals. The reservoir temperatures estimated by multi-geothermometries have a range of 63–150 °C for shallow geothermal water and of 190–210 °C for deep geothermal water, respectively. The δ^18^O and δD compositions indicated geothermal waters are recharged by meteoric water from the elevation of 2923–5162 m. Based on the aforementioned analyses above, a conceptual model was constructed for the geothermal system in the Xianshuihe fault zone.

## 1. Introduction

Nowadays, energy shortages and environmental pollution are becoming more and more serious and therefore sustainable development is hampered. To address the aforementioned problems, the exploitation and utilization of clean energy are urgently required. Geothermal resources have become a popular clean energy source due to its clean and renewable affinities [1]. Geothermal springs, as an important constituent of geothermal resources, have become a hot research topic so far [2,3,4,5,6].

**Figure 1:**
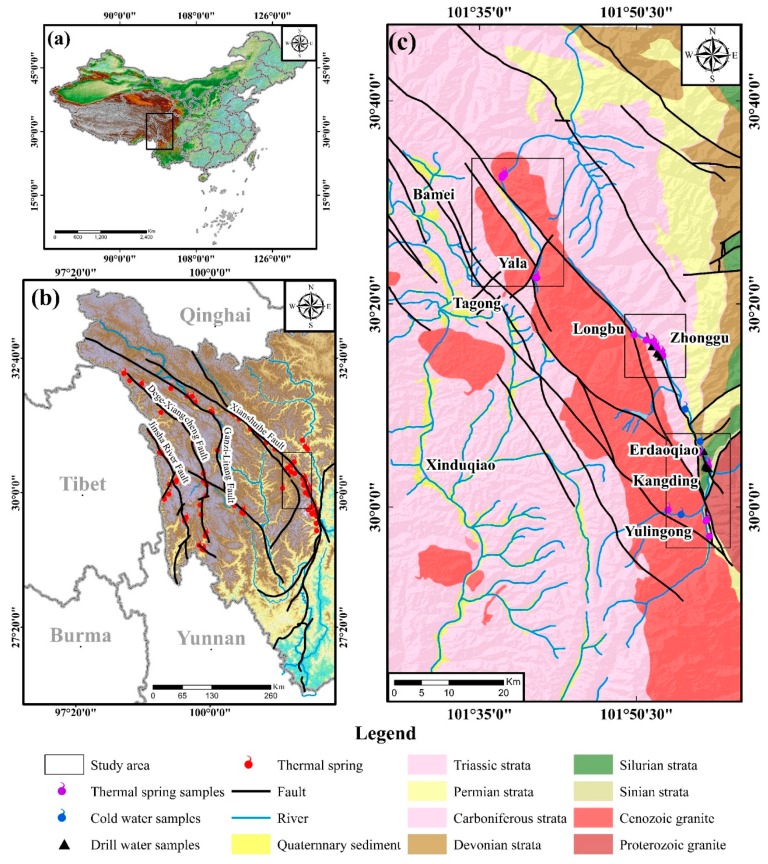
(**a**) Location of western Sichuan in China, (**b**) the distribution of geothermal springs in western Sichuan, and (**c**) the distribution of geothermal springs in the Xianshuihe fault zone.

Abundant geothermal springs have been reported on the Chinese mainland [7]. Most of the high-temperature geothermal springs are distributed in southwestern China, including southern Tibet, western Sichuan, and western Yunnan [8,9,10,11,12,13,14]. The Xianshuihe fault zone (XFZ) of western Sichuan is a famous area with a significant number of geothermal springs [15] (Figure 1a,b). Geothermal springs are primarily found in the Yalabamei, Zhonggu, Erdaoqiao, and Yulingong geothermal areas of the XFZ (Figure 1c). Up to now, previous studies have interpreted the genesis of geothermal springs in the Erdaoqiao and Yulingong geothermal areas using geophysical and geochemical methods [16,17,18,19,20,21,22,23,24]. However, only a few previous investigations analyzed the geothermal springs in the Yalabamei and Zhonggu geothermal areas [25], and thus the conceptual model of the geothermal system is unclear. Further research has yet to be conducted to facilitate the exploitation and utilization of the geothermal resource in the XFZ.

Therefore, this study aims at clarifying the mechanism of the geothermal system in the XFZ. Forty-one geothermal springs, drilled water wells, and cold water sites were sampled in the Yalabamei, Zhonggu, Erdaoqiao, and Yulingong geothermal areas of the XFZ. Hydrogeochemical and D-O isotopic analyses for those samples were employed to trace the water–rock interaction, reservoir temperature, and recharge source of geothermal water. Afterward, the conceptual model of the geothermal system was preliminarily constructed as a summary of our hydrogeochemical analyses.

## 2. Study Area

The XFZ is located in southwestern China and tectonically belongs to the eastern margin of the Tibetan Plateau. Since the Eurasian-Indian collision at the Eocene, the XFZ has been moving in the way of the left-lateral strike-slip [26]. The XFZ is composed of northwestern, middle, and southeastern segments. The northwestern segment includes the Luhuo, Daofu, and Bamei faults. The middle segment consists of the Yalahe, Zheduotang, and Selaha faults. The southeastern segment contains the Kangding and Moxi faults. The geomorphology of the XFZ is high mountain and low valley. The elevations of the XFZ have a range of ~3000–7556 m with a significant difference of 3000–4000 m.

In the study area, the sedimentary strata are dominated by Triassic sandstone and slate with minor Proterozoic marble, schist, and phyllite, Sillure schist and marble, Devonian slate, Permian limestone, and Quaternary sediments. Three periods of magmatic events (the Proterozoic, and early and late Yashanian) have been recognized in the study area. Separated by the XFZ, the Proterozoic igneous rocks are exposed on the eastern side, while early and late Yanshanian igneous rocks are emplaced on the western side.

An abundance of geothermal springs has been investigated in the middle and southeastern segments of the XFZ, heated by deep magma, radioactive heat of granitoids, and strike-slip frictional heat of the XFZ [15]. Their distributions are well controlled by the XFZ, forming the Yalabamei, Zhonggu, Erdaoqiao, and Yulingong geothermal areas (Figure 1c). (1) the Yalabamei area: Twenty geothermal springs with measured temperatures of 47–66 °C are exposed in the Yanshanian granite. They are mainly distributed along the northeastern-trending faults. (2) The Zhonggu area: More than one hundred geothermal springs have been reported in the Zhonggu area. They are distributed along the Yalahe fault and exposed in the Triassic sandstone. The flow rate ranges from 0.1 to 0.3 L/s and the highest temperature is 43 °C. (3) The Erdaoqiao area: The geothermal springs with a discharge of 0.5 to 6.4 L/s and a temperature of 32–56 °C are exposed in the Quaternary sediments along the Yalahe fault. A large number of travertines have been observed on the surface, as well as a strong H_2_S smell. (4) The Yulingong area: The geothermal springs here have a temperature higher than 60 °C (the highest is 94 °C) and the discharge of 0.24–6.34 L/s.

## 3. Sampling and Methodology

A total of 36 water samples were collected from geothermal springs, geothermal drilled water wells, and cold springs and rivers in the vicinity of the XFZ on 18–20 May 2017. Field sampling procedures and analytical methodology were described by Zhang et al. (2018) [16]. Temperature, pH, alkalinity, and total dissolved solids (TDS) were measured in the field. Temperatures of the samples were measured using a mercury thermometer. The pH electrode was calibrated with standard solutions of pH 1.0, 4.0, and 7.0. The alkalinity of the samples was measured by Gran titration with 0.1 M HCl and is expressed here as HCO_3_^−^. Samples for cation analysis were filtered through 0.1μm and acidified to pH less than 1 with HNO_3_. Anions were determined within 24 h after collection.

Within a week after fieldwork, all the experiments were carried out in the State Key Laboratory of Geohazard Prevention and Geoenvironment Protection, Chengdu University of Technology. The samples were analyzed for Si and major cations (K, Na, Ca, and Mg) using inductively coupled plasma-optical emission spectrometry (ICP-OES) (Thermo Fisher ICAP-6300), while anions (Cl and SO_4_) were determined by ion chromatography (Dionex ICS-1100). Charge balance errors between major cations and anions were lower than ±10% for all samples. The δD and δ^18^O values were reported in delta (δ) relative to VSMOW (Vienna Standard Mean Ocean Water) using conventional δ (‰) notation. The analytical precision for δD and δ^18^O was ± 0.6‰ and ± 0.2‰, respectively.

## 4. Analytical Results

**Figure 2:**
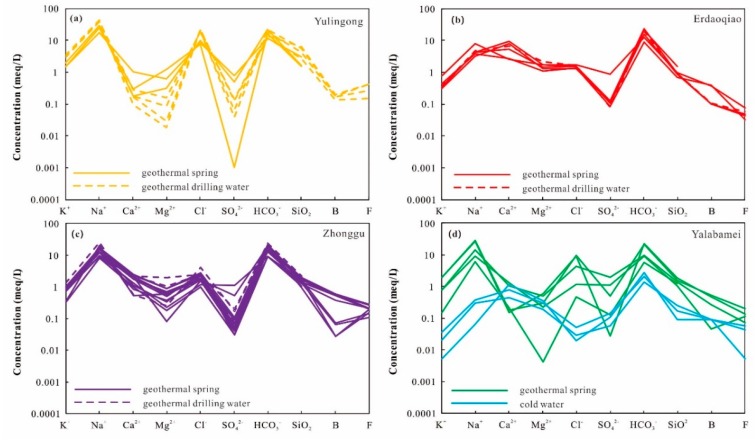
Schoeller semilogarithmic diagram of geothermal water samples from the (**a**) Yulingong area, (**b**) Erdaoqiao area, (**c**) Zhonggu area, and (**d**) Yalabamei area, with cold water samples here instead of geothermal drilling water.

**Figure 3:**
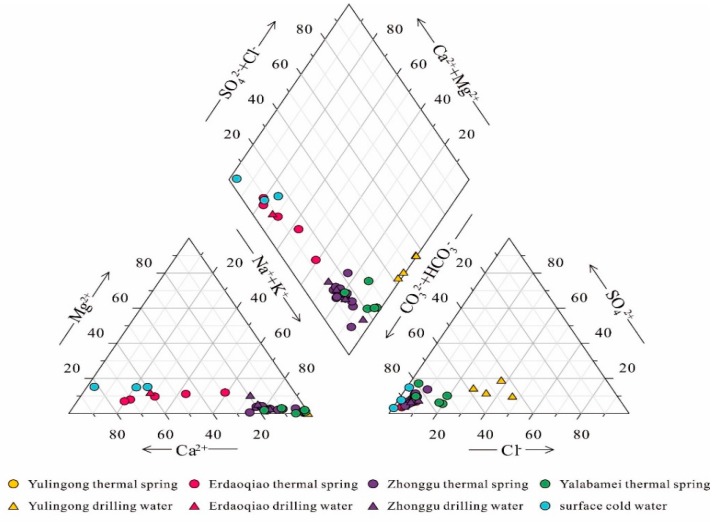
Piper plots of selected cold waters and geothermal waters in the Xianshuihe fault zone (XFZ) [27].

The physical properties and chemical compositions of geothermal waters along the Xianshuihe fault are presented in Table S1. The exposed temperature values range from 30.5 to 115 °C, and the pH values vary from 6.5–9.0. Cold spring and river (referred to as cold water afterward) samples have temperature values of 9.8–12.8 °C and pH values of 7.9–8.1. Na^+^ and Ca^2+^ are the primary cations, while HCO_3_^−^ and Cl^−^ are the dominant anions, respectively (Figure 2). According to the major-ion characteristics of geothermal waters, four representative groups of hydrochemical types were recognized, as shown in Figure 3. The Na–HCO_3_–Cl type geothermal waters are exposed in the Yulingong area and possess higher TDS and temperature values. The Ca–Na–HCO_3_ type geothermal waters are distributed in the Erdaoqiao area, while Na–HCO_3_ type geothermal waters are located in other areas. Both of the Ca–Na–HCO_3_ and Na–HCO_3_ type geothermal waters are relatively lower TDS and temperature values. Cold waters are of the Ca–HCO_3_ type with the main ions of Ca^2+^ and HCO_3_^−^ (Figure 3).

The concentrations of SiO_2_, B, and F do not display significant variation among the above four types of geothermal waters (Figure 2). The SiO_2_ concentrations vary from 42.0 mg/L to 367.1 mg/L. The highest SiO_2_ concentrations were observed from the Yulingong drilling holes. The F concentrations had a range from 0.6 mg/L to 10 mg/L. The boron concentration (B) are from 0.3 mg/L to 6.6 mg/L. The small variations of boron concentration imply minor involvements of magmatic composition.

The δ^18^O and δD compositions (vs. Vienna-Standard Mean Ocean Water (VSMOW)) of the sampled waters vary from −9.3‰ to −11.4‰ and from −63.1‰ to −75.0‰, respectively (Supplementary Material: Table S1). The waters in the borate exploration wells were slightly depleted in these two isotopic compositions.

## 5. Discussion

### 5.1. Processes Controlling the Major Ionic Compositions

#### 5.1.1. Correlations of Major Ions

**Figure 4:**
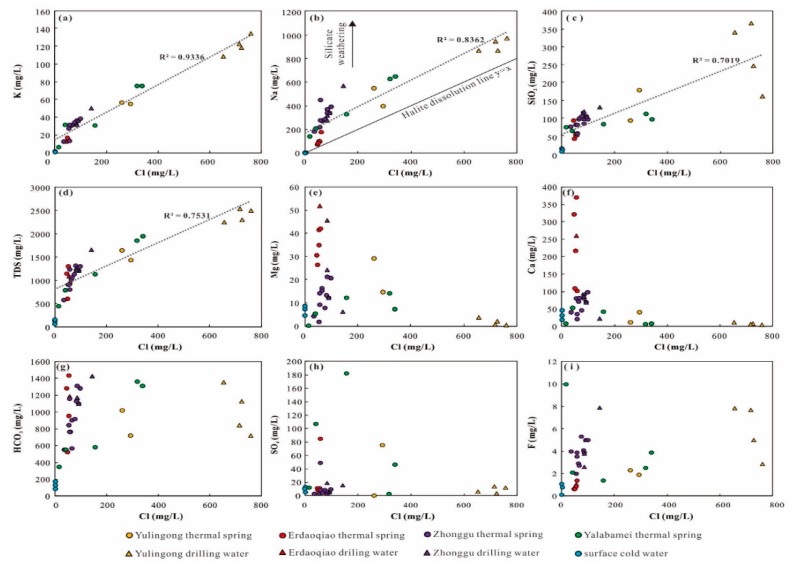
The plots of Cl against (**a**) K, (**b**) Na, (**c**) SiO_2_, (**d**) TDS, (**e**) Mg, (**f**) Ca, (**g**) HCO_3_, (**h**) SO_4_, and (**i**) F.

Among these major ions, Cl used to be employed as a useful tool to trace the geochemical process due to its conservative affinity. Even in the condition of high temperatures and high pressures, the Cl concentration would hardly be altered by water–rock interactions and adsorption of rock-forming minerals. Therefore, the relationship between the Cl and other major ions is feasible to clarify the hydrochemical processes in the circulation of geothermal waters. In Figure 4, geothermal drill samples are generally around or higher than geothermal spring samples, while cold water samples concentrated around zero with no linear trend. It is observed that Cl are well correlated with K (squared regression coefficients = 0.9336) (Figure 4a). Geothermal waters are believable to be mixing products between surface cold water and a deep geothermal fluid. Linear relationships between Na or SiO_2_ and Cl concentrations also existed in (Figure 4b,c). However, in comparison, squared regression coefficients (0.8362 and 0.7019) of Na or SiO_2_ vs. Cl are obviously lower than that for K vs. Cl. Considering this, Na and SiO_2_ would also be derived from the mixture but may be affected by other processes (e.g., water–rock reaction and ion exchange). The plots of geothermal spring samples above the line of halite dissolution further suggest Na and Si were originated from silicate dissolution (Figure 4a).

In addition, the plots in the Ca, Mg, HCO_3_, SO_4_, and F vs. Cl diagrams present scattered distributions (Figure 4e–i). Hence, these ions are suggested to be derived from multiple sources. When the Ca^2+^/HCO_3_^−^ and (Ca^2+^ + Mg^2+^)/HCO_3_^−^ molar ratios are equal to 0.5, those ions are attributed to the dissolution of calcite and dolomite (Equations (1) and (2)).

CaCO_3_ (calcite) + H_2_CO_3_ → Ca^2+^ + 2HCO_3_^−^ (1)

CaMg(CO_3_)_2_ (dolomite) + 2H_2_CO_3_ → Ca^2+^ + Mg^2+^ + 4HCO_3_^−^ (2)

**Figure 5:**
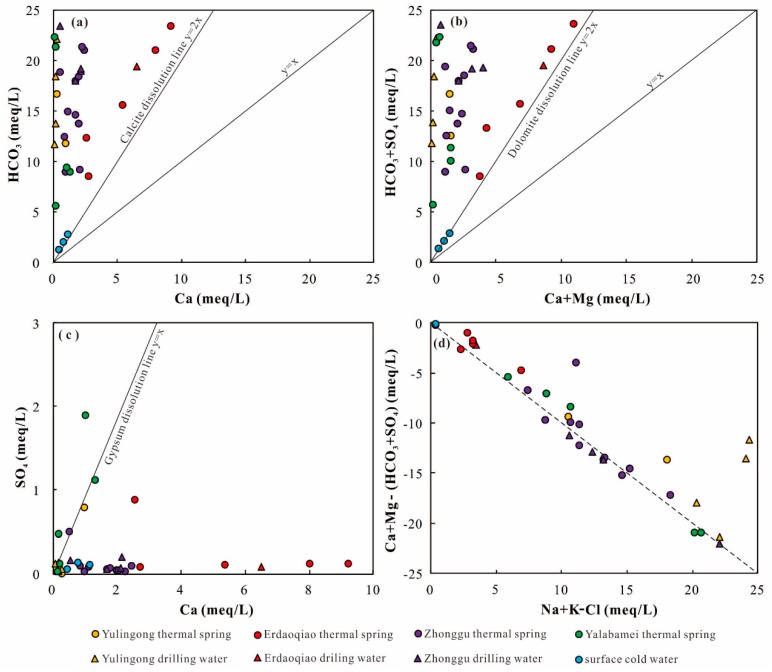
Diagrams used to identify hydrogeochemical processes based on the concentrations of major constituents in water. (**a**) Ca vs. HCO_3_, (**b**) Ca + Mg vs. HCO_3_ + SO_4_ (**c**) Ca vs. SO_4_, and (**d**) Na + K − Cl vs. Ca + Mg − (HCO_3_ + SO_4_).

In Figure 5a, the Ca^2+^/HCO_3_^−^ and (Ca^2+^ + Mg^2+^)/HCO_3_^−^ molar ratios are lower than 0.5. The low Ca^2+^ and enrichment of HCO_3_^−^ are attributed to ion exchange from silicates dissolution.

When the Ca^2+^ and SO4^2−^ are derived from dissolution of gypsum, the ratio between Ca^2+^ and SO_4_^2−^ would be 1:1 (Equation (3)).

CaSO_4_·2H_2_O ⇄ Ca^2+^ + SO_4_^2−^ + 2H_2_O (3)

In this study, most of the samples are plotted distinctly below 1:1 line in Ca^2+^ versus SO_4_^2−^ diagram (Figure 5c), indicating the significantly higher concentration of Ca^2+^. Hence, the enriched Ca^2+^ would be produced from the dissolution of carbonates and silicate minerals.

In the (Na + K) − Cl and (Ca + Mg) − (SO_4_ + HCO_3_) diagram, most of the samples are plotted along the 1:1 line. Hence, the hydrochemical composition of the samples are controlled by a cation-exchange process that is the result of silicate dissolution (Figure 5d).

#### 5.1.2. Principle Component Analysis

**Figure 6:**
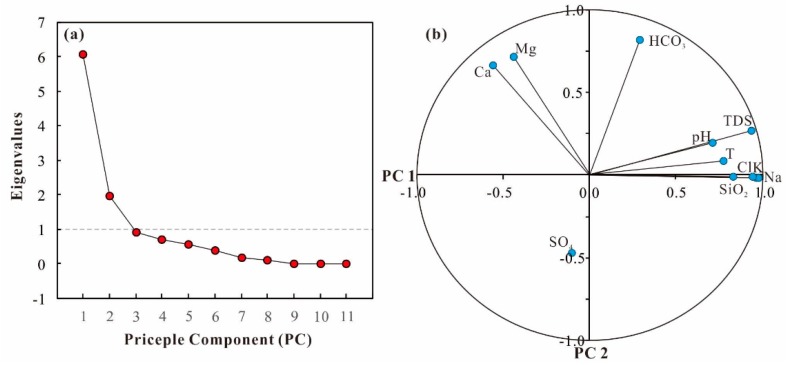
Diagrams of principle component analysis, (**a**) scree plot, (**b**) factor loadings for PC1 and PC2.

Hydrochemical parameters including pH and major ions were used for principal component analysis, which is helpful for tracing the sources of those ions [16,17]. The results of the principal component analysis include eigenvalue, percentage of variance, the cumulative percentage of variance, and the factor loading, presented in Table 1. Scree plots for groundwater samples showed a distinct change of slope after the second factor (Figure 6a). Using the Kaiser Criterion and scree plot, two principal components (PCs) of eigenvalues greater than 1 have been obtained, accounting for a total variance of 73.084%. The PC1 was responsible for 55.250% of the total variance and has a strong loading of TDS, Na^+^, K^+^, Cl^−^ and SiO_2_ (Figure 6b). This factor indicates the general trend of hydrochemical characteristics, probably dominated by the mixture between cold surface water, deep geothermal water, and the dissolution of silicate minerals. The PC2 explained 17.835% of the total variance and has a medium positive loading of Ca^2+^, Mg^2+^, and HCO_3_^−^ (Figure 6b). As such, PC2 could be linked to the dissolution of limestone and dolomite.

Based on the analyses of correlations of major ions and the principal component analysis, it is possible to constrain the sources of different water types in the XFZ. The Na–HCO_3_–Cl type geothermal waters would be derived from the dissolution of silicate rocks with the mixing of deep fluids. Na–HCO_3_ type geothermal waters are the products from the dissolution of silicate rocks, while Ca–Na–HCO_3_ type geothermal waters are originated from the dissolution of silicate and carbonate rocks.

### 5.2. Geothermometry

Geothermometers are used to estimate reservoir temperatures for most systems. The geothermometers are based on temperature-dependent, water–rock equilibria that control the chemical and isotopic composition of geothermal waters. The temperature of the reservoir associated with the geothermal system of the XFS was estimated using both classical geothermometers (cation and silica), the silicon-enthalpy graphic method, and geothermometrical modeling.

#### 5.2.1. Classical Geothermometry

**Figure 7:**
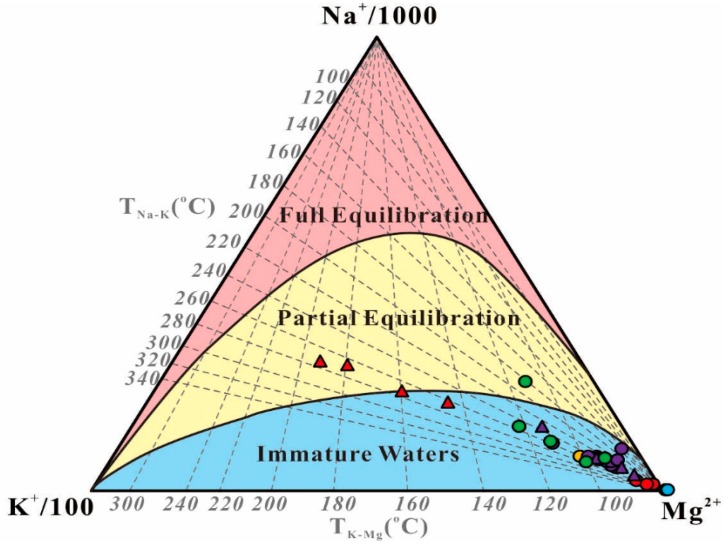
Na–K–Mg trilinear equilibrium diagram of the geothermal waters in the XFZ [28]. Legends are followed by those in Figure 5.

The classic chemical geothermometers (e.g., cation and silica) are applicable for the estimation of the equilibrium temperature in geothermal reservoirs. Considering this, cation and silica geothermometers were conducted to calculate the equilibrium temperatures as listed in Table S1. However, the results from the cation geothermometers have a great range and have a large variation with wellhead temperatures. In the Na–K–Mg ternary diagram, most of the samples are located in the area of immature water, whereas only the samples of the Yulingong deep wells are found in semi-mature fields (Figure 7). The diagram demonstrates that none of the geothermal spring waters have reached full equilibrium with the host. Therefore, Silica geothermometers are more applicable to the geothermal spring waters than cation geothermometers in this study [28].

**Figure 8:**
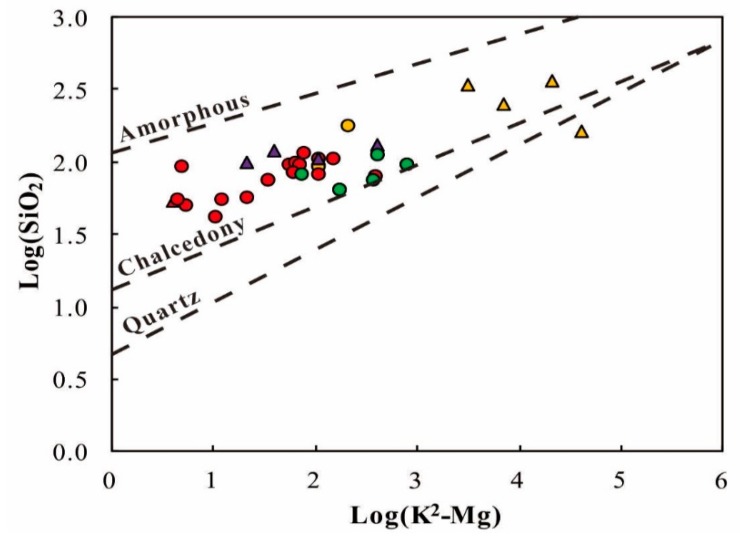
Cross plots of log (K^2^/Mg) vs. log(SiO_2_) of the geothermal waters in the XFZ [29]. Legends follow those in Figure 5.

Silica geothermometry is based on the solubility of different silica species (mostly e.g., quartz, chalcedony) in water as a function of reservoir temperature. In Table S1 (see Supplementary Material) estimated reservoir temperatures in the study area using various silica geothermometers are presented. The plots in the log (K^2^/Mg) versus the log(SiO_2_) diagram shows most of the samples are distributed above the chalcedony curve (Figure 8). The chalcedony saturation indices higher than zero indicate the chalcedony is oversaturated. In addition, quartz geothermometers are used for relatively high temperatures, but at temperatures lower than 180 °C, chalcedony may control the dissolved silica concentration in geothermal fluids. The estimated reservoir temperatures are mostly lower than 180 °C. Consequently, the chalcedony geothermometer is the most appropriate silica geothermometer to estimate reservoir temperatures of the geothermal springs, yielding the results from 63–150 °C.

#### 5.2.2. Silicon-Enthalpy Graphic Method

Due to the immature affinity of geothermal waters, the mixing of cold water should be considered in the calculation of reservoir temperature [30]. As such, it is reliable to use for estimating the reservoir temperature of mixed geothermal water. In this study, cold and geothermal water samples are plotted in the silica enthalpy mixing model and the silica concentration and corresponding enthalpies are determined by the international steam tables [31].

**Figure 9:**
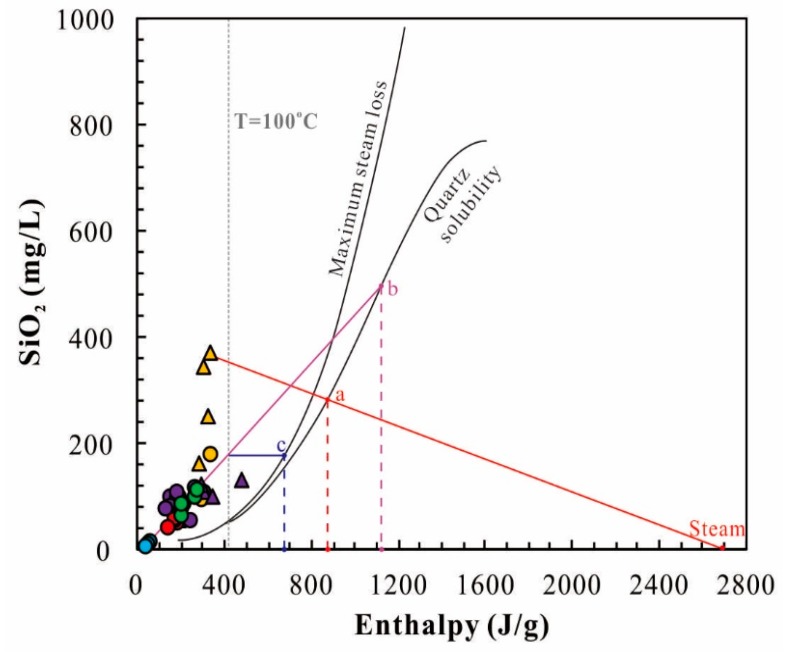
The silica-enthalpy plot of the geothermal waters and cold waters in the XFZ. Legends follow those in Figure 5.

Figure 9 presents the silica-enthalpy mixing model according to chalcedony and quartz solubilities. Two end-member fluids have been given in this model: the cold water sample as one end member and the geothermal waters as the other end member. A red line linking deep geothermal well water and steam point intersected with the Quartz solubility line at the point a. The point stands for the enthalpies/temperatures for the deep. A purple line was drawn from cold water to the geothermal springs and intersected with the Quartz solubility line at the point b. The horizontal axis of the point b is the reservoir temperature in the condition of no steam separation before mixing. A horizontal blue line is drawn at the intersection between the purple line and the vertical line of the boiling point of water intersected with the maximum steam loss line at point c. The horizontal axis of the point c is the reservoir temperature in the condition of steam separation occurs before mixing. Based on the aforementioned above, the estimated reservoir temperatures of deep geothermal well water and geothermal springs are 208 °C, 265 °C (no steam separation before mixing), and 164 °C (maximum steam loss before mixing), respectively. It is noted that the temperature of deep geothermal well water is lower than that of the geothermal spring. This phenomenon would be attributed to CO_2_ degassing that leads to the SiO_2_ reprecipitate. The reservoir temperatures (63–150 °C) estimated by the chalcedony geothermometer are compatible with the temperature (164 °C) in the condition of maximum steam loss before mixing. Therefore, the reservoir temperature determined by the silica-enthalpy mixing model is approximately 164 °C when no steam separation occurred before mixing. In addition, the mixture ratio of cold water can be estimated by the length of point b and the end-member of the geothermal spring against the length of point b and the end-member of cold water (Figure 9). As such, the mixture ratios of cold water are about 70–90%.

#### 5.2.3. Geothermometrical Modeling

**Figure 10:**
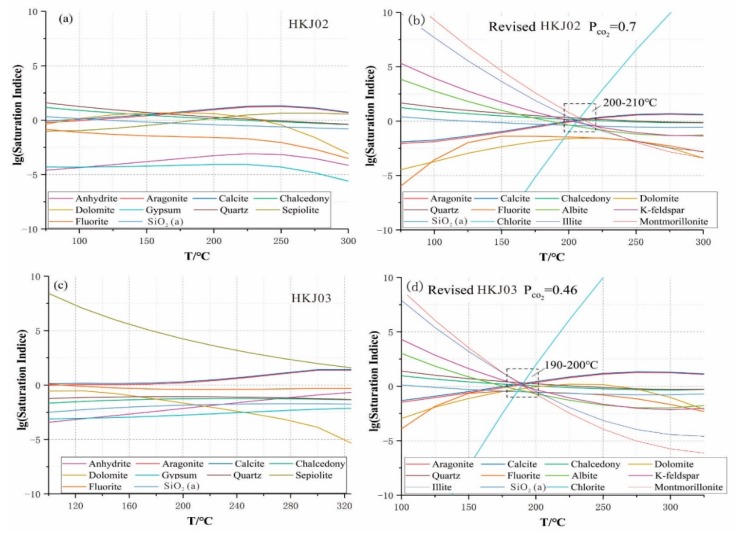
Initial and reconstructed mineral equilibria graph of representative drilling water samples HKJ02 (**a**,**b**) and HKJ03 (**c**,**d**).

A multi-mineral saturation geothermometer is employed to estimate reservoir temperature when the geothermal water in the reservoir reaches mineral equilibrium [32]. In this study, the variations of the saturation indices of different mineral temperatures were calculated at a temperature step of 20 °C using SOLVEQ-XPT. The two samples with the highest wellhead temperatures (HKJ02 and HKJ03, see in Table S1) are taken as the representatives for the Yulingong area. To correct CO_2_ degassing and possible aluminum concentration error, the calcite and microcline were forced to reach mineral equilibrium. For the geothermal wells (HKJ02 and HKJ03), saturation indices (SI) with respect to albite, aragonite, calcite, chalcedony, dolomite, quartz, laumontite, fluorite microcline, K-feldspar, illite, SiO_2_(a), chlorite, and montmorillonite minerals tend to get closer to zero (SI = 0) around the temperature of 200–210 °C and 190–200 °C (Figure 10). The temperature range indicates the estimated reservoir temperature at which these minerals reach an equilibrium condition. Meanwhile, the estimated reservoir temperatures estimated by the multi-mineral saturation geothermometer are consistent with the estimated reservoir temperature (208 °C) obtained by the silicon-enthalpy graphic method.

### 5.3. Recharge Origin Traced by δD and δ^18^O

**Figure 11:**
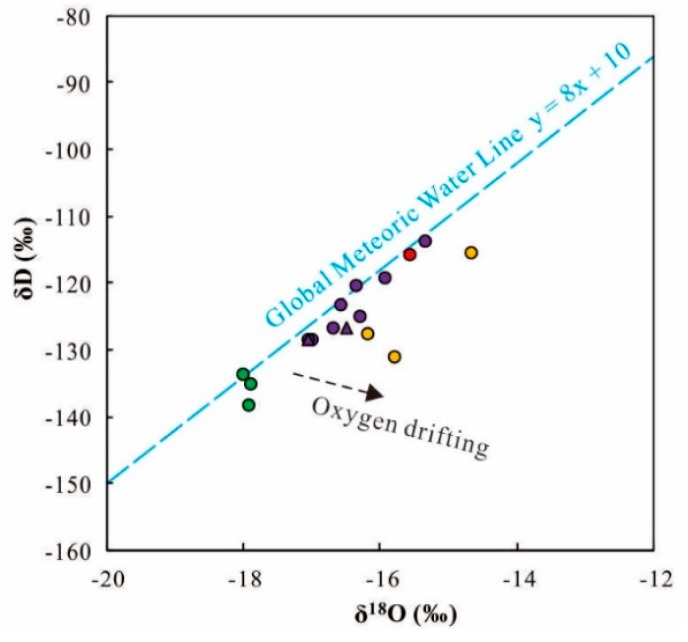
Plot of δ^18^O–δD for the geothermal waters in the XFZ. Global meteoric water line (GWML) is after [33]. Legends follow those in Figure 5.

H and O stable isotopes (δD and δ^18^O) are useful to trace the recharge origin of surface and ground waters. In this study, the δD and δ^18^O values of geothermal waters range from −138.5‰ to −113.8‰ (average = −125.9‰) and −18.0‰ to −14.7‰ (average = −16.5‰), respectively (Table S1). The geothermal waters are plotted close to the Global Meteoric Water Line (GMWL) [33] in the δD–δ^18^O graph, suggesting recharge origin of meteoric water (Figure 11). It is noteworthy that the plots of geothermal waters are slightly deviated from the global meteoric water line (GMWL), representing the occurrence of oxygen-isotope drifting. This phenomenon may be attributed to the oxygen isotope exchange by the water–rock reaction between geothermal waters and the surrounding carbonate or silicate rocks (calcite or silicate).

Due to the altitude effect of δD and δ^18^O, they can be used to calculate the recharge elevation. Considering the existence of oxygen drifting, the δD values of the geothermal waters are more robust to estimate the recharge elevation in this study. The recharge elevations of geothermal water are calculated based on Equation (4) below [33]:

H = h + (δS − δP)/K(4)

where H is the recharge elevation (m), h is the reference point elevation, δS is the δD or δ^18^O value of sampled geothermal waters, δP is the δD or δ^18^O value of recharge water, and K is the δD or δ^18^O elevation gradient of atmospheric precipitation (δ/100 m). In this study case, h is 270 m, δP is −52.9‰ for δD and −7.0‰ for δ^18^O, and K is −1.12‰/100 m for δD and −0.26‰/100 m for δ^18^O [34]. Accordingly, the recharge elevation of the geothermal waters in the XFZ are calculated as 2923–5162 m (Table S1).

### 5.4. Conceptual Model

**Figure 12:**
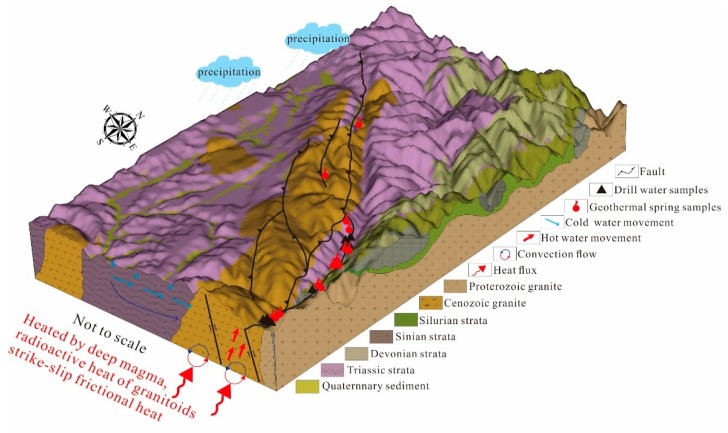
Preliminary conceptual model of the geothermal system in the XFZ.

Based on the available geochemical, structural, and hydrogeological data, the following conceptual model is proposed, integrated in Figure 12.

A previous study proposed the geothermal system of the XFZ is a liquid-dominated system heated by deep magma, radioactive heat of granitoids, and strike-slip frictional heat. D-O isotopes indicate geothermal water is recharged by meteoric water with precipitation elevation of 2923–5162 m and then travel along the developed faults and fractures of the XFZ. Water–rock is common in the circulation of geothermal water because of obvious δ^18^O drifting. The major ions (e.g., Na^+^, Ca^2+^ and HCO_3_^−^) of geothermal water are derived from the dissolution of silicate and carbonate minerals and ion exchange. Shallow geothermal water with Ca–HCO_3_ or Na–HCO_3_ types is produced by the mixture between Na-Cl-HCO_3_ type deep geothermal water and Ca–HCO_3_ surface cold water. Deep and shallow geothermal waters possess the reservoir temperatures of 63–150 °C and 190–210 °C, respectively. No steam separation occurred before mixing. Finally, geothermal water arises and emerges as geothermal spring in the area where faults and fractures exist, mixed by 70%–90% cold water.

## 6. Conclusions

The physical and chemical processes controlling the chemical composition of the geothermal waters in the XFS were investigated. In this context, the results obtained from geochemical and isotopic studies are listed below.

1. Shallow geothermal waters represented by geothermal springs and shallow drilled wells are divided into two hydrochemical groups: (1) Ca–Na–HCO_3_ type in the Erdaoqiao area, and (2) Na–HCO_3_ in other areas. Deep geothermal waters represented by deep drilled wells are characterized by Na–Cl–HCO_3_ type. Cold waters are of the Ca–HCO_3_ type.
2. Correlations of major ions and principle component analyses agree that the major ionic compositions of geothermal water are primarily determined by the water-rock interaction with silicate and carbonate minerals.
3. Silica and silicon enthalpy graphic method and geothermometrical modeling yield the reservoir temperatures of 63–150 °C for shallow geothermal waters, and of 190–210 °C for shallow geothermal waters, respectively. Cold water at 70–90% was proposed to be mixed with geothermal water.
4. The δ^18^O and δD compositions indicated geothermal waters are recharged by meteoric water from the elevation of 2923–5162 m. Oxygen drifting implies the occurrence of water–rock interaction in the formation of geothermal waters.

## Figures and Tables

**Table 1:** Factor loadings and eigenvalues of the eleven extracted factors.

Variables	PC1	PC2
K	0.9653	−0.0195
Na	0.9840	−0.0203
Ca	−0.5575	0.6614
Mg	−0.4390	0.7169
Cl	0.9452	−0.0088
SO_4_	−0.0993	−0.4763
HCO_3_	0.2919	0.8155
T	0.7142	0.1942
pH	0.7764	0.0826
TDS	0.9387	0.2700
SiO_2_	0.8315	−0.0133
Eigenvalue	6.0770	1.9620
Variance (%)	55.25	17.835
Cumulative (%)	55.25	73.0840

## Supplementary Materials

The following are available online at https://www.mdpi.com/1660-4601/17/2/500/s1, Table S1: Hydrochemical and δ^18^O and δD compositions of geothermal and cold waters in the Xianshuihe Fault Zone.
